# Will coronavirus disease (COVID-19) have an impact on antimicrobial resistance?

**DOI:** 10.2807/1560-7917.ES.2020.25.45.2001886

**Published:** 2020-11-12

**Authors:** Dominique L Monnet, Stephan Harbarth

**Affiliations:** 1 European Centre for Disease Prevention and Control (ECDC), Stockholm, Sweden; 2 Infection Control Program and Division of Infectious Diseases, University of Geneva Hospitals and Faculty of Medicine, Geneva, Switzerland

**Keywords:** COVID-19, bacterial infections, viral infections, respiratory viruses, antibiotic use, antimicrobial resistance, hygiene, infection control, surveillance, automated surveillance

Since the beginning of 2020, the ongoing coronavirus disease (COVID-19) pandemic has resulted in the deaths of more than 250,000 patients in the European Union/European Economic Area (EU/EEA) and the United Kingdom alone [1]. By the end of 2020, as every year, bacterial infections with antimicrobial resistance (AMR) will have caused the deaths of more than 30,000 Europeans [2]. AMR is another ongoing pandemic that often goes unnoticed by a majority of Europeans. Larry Kerr, co-chair of the Transatlantic Task Force on Antimicrobial Resistance, recently compared the AMR pandemic to a multitude of small fires that are much less visible than the single massive firestorm that is the COVID-19 pandemic.

While experts have warned of the link between COVID-19 and AMR [3-8], studies report conflicting evidence. Several studies—from, in particular, Germany, Italy and the US—have reported outbreaks or an increase in infections with and/or acquisition of multidrug-resistant bacteria during the COVID-19 pandemic [9-12]. Further studies have reported cases of antimicrobial-resistant invasive fungal infections in COVID-19 patients [13,14], and one case of azole-resistant *Aspergillus* spp. infection in an immunocompetent COVID-19 patient [15]. However, other studies from France and Spain did not show an increase in infections with multidrug-resistant bacteria [16,17], and one Italian study even saw a reduction in *Clostridioides difficile* infections in hospitalised patients [18]. In a rapid review, Fattorini et al. found that only 1.3% of 522 COVID-19 patients in intensive care units (ICUs), and apparently no COVID-19 patients in other units, developed a healthcare-associated superinfection with antimicrobial-resistant bacteria [19].

These different experiences may just be the consequence of previous antibiotic prescribing and infection prevention and control (IPC) practices that resulted in varying background AMR prevalence, in particular in healthcare settings, in different countries [8,20]. Still, antibiotic prescribing and IPC practices may change during the COVID-19 pandemic and AMR could either increase or decrease as a result of these changes [5]. As pointed out by Nieuwlatt et al. [21], COVID-19 and AMR are parallel and interacting health emergencies that have similarities and offer opportunities for mutual learning with regard to their control. But the question remains whether the AMR situation in Europe and elsewhere will worsen or improve as a consequence of the current COVID-19 pandemic.

We summarised various determinants that may result in either an increase or, inversely, a decrease in AMR and found them to be balanced (Table). The truth is that the impact of the COVID-19 pandemic on AMR will only become clear in the coming months and years as data gradually become available. Changes in AMR will most likely vary depending on the setting—e.g. ICUs vs other hospital units, hospital vs community settings—and possibly between countries.

**Table:** Factors that may influence levels of antimicrobial resistance during the COVID-19 pandemic

Type of factor	Factors that may favour an increase in AMR	Factors that may favour a decrease in AMR
Antibiotic use in hospitals	• About 70% of hospitalised COVID-19 patients receive antibiotics [33,34] • COVID-19 patients often receive empiric broad-spectrum antibiotic therapy [34-36] • 16% of hospitalised COVID-19 patients develop a secondary bacterial infection [34], which will necessitate antibiotic therapy • Possible increased use of azithromycin and teicoplanin (because of the initial absence of clear guidelines for the treatment of COVID-19 patients) [4,6,8] • Difficulties in accessing advice from experts before prescribing antimicrobial agents [4] • Antimicrobial stewardship efforts may be undermined because of high workloads and shifting priorities related to COVID-19 [37,38] • Possible aggravation of existing shortages of certain narrow-spectrum antimicrobial agents [39,40]	• Bacterial co-infection (estimated on presentation) in only 3.5% (95% CI: 1–7%) of COVID-19 patients [33] • Bacterial/fungal infection in only 8% of hospitalised COVID-19 patients vs 11% in non-COVID-19 patients [34]; the percentage for COVID-19 patients may be underestimated because many may have received empiric antimicrobial therapy [41] • Only 1.3% of COVID-19 patients in ICUs, and apparently no patients in other units, developed a healthcare-associated superinfection with antimicrobial-resistant bacteria [19] • Postponed planned surgical interventions result in fewer antibiotic courses for surgical prophylaxis [42] • Fewer emergency and planned hospital admissions [43,44], including chronically ill patients (e.g. oncology patients, diabetic patients, transplant patients), resulting in fewer antibiotic prescriptions
Infection prevention and control in hospitals	• Difficulties for HCWs in adhering to standard IPC precautions because of long shifts wearing the same PPE [45] and possible shortages of certain equipment [5] • Focus of HCWs on self-protection (e.g. universal gloving practices) rather than on preventing cross-transmission between patients • In COVID-19 cohort units and ICUs, sessional use of PPE, e.g. long-sleeved gowns that prevent effective hand hygiene [46] and gloves that may not be changed between patients [45] • Overcrowded facilities and possible staff shortages leading to low HCW-to-patient ratios [5] • Shortages of HCWs with appropriate IPC training [4] • Longer hospital stays for COVID-19 patients [5] • Traditional IPC efforts may be temporarily discontinued, including those targeting antibiotic-resistant bacteria, e.g. decreased frequency of screening for carriage of MDROs and difficulties in isolating or cohorting MDRO-positive patients [4,47] • Decreased laboratory capacity to detect AMR carriage, e.g. for processing rapid tests for MDROs, because resources are focused on SARS-CoV-2 diagnosis [4]	• Isolation of COVID-19 patients with enhanced standard precautions, e.g. increased hand hygiene and use of PPE, plus universal chlorhexidine bathing protocols for patients in ICUs [5] • Increased disinfection of the environment [4,5] • COVID-19 patients are often cohorted in one single unit and cared for by the same group of HCWs [5] • Fewer emergency and planned hospital admissions [43,44], including chronically ill patients (e.g. oncology patients, diabetic patients, transplant patients), resulting in lower colonisation pressure by fewer carriers of MDROs • Fewer transfers from long-term care facilities may lead to fewer cycles between long-term care facilities and hospitals [5] • Construction of new COVID-19 facilities without an established reservoir of MDROs [5]
Antibiotic use in the community	• Likely increased antibiotic use in nursing homes and other long-term care facilities • Possible increased self-medication with antibiotics in some countries or regions of the world [48]	• Possible decreased antibiotic consumption because of fewer patient consultations, e.g. for self-limiting infections that would otherwise have resulted in an antibiotic prescription [4] • Possible decreased incidence of respiratory tract infections as a consequence of decreased person-to-person transmission because of lockdowns, resulting in decreased antibiotic consumption • Possible increased awareness of the difference between viruses and bacteria, and the fact that there are different types of medicines, i.e. antivirals and antibiotics, respectively, for different types of infections [8] • Increased influenza vaccine uptake may decrease the incidence of bacterial superinfections after influenza
Hygiene practices in the community	• Increased use of sanitisers and other biocidal agents and their release in the environment [3,6,8,49]	• Increased hand hygiene practices and compliance in the community • Increased physical distancing and use of face masks • Increased disinfection of the environment
Cross-border spread		• Fewer patient transfers of seriously ill patients between countries, resulting in less frequent cross-border spread of MDROs • Large decrease in international air travel, resulting in decreasing risk of global dissemination of antimicrobial-resistant bacteria and genes from highly endemic regions [8,50]
Public health policy making, including One Health	• Shift in high-level policy making towards viral diseases and preparedness for emerging viruses • National plans and other initiatives to fight AMR are likely to have been slowed down, temporarily discontinued or even postponed because of COVID-19 public health emergencies and duties (similar to the WHO Global Strategy for Containment of Antimicrobial Resistance, which was launched on 11 September 2001 and went largely unnoticed by the global community, without any major impact on AMR activities for almost a decade, because of the disproportional focus on biosecurity issues) • Potential One Health impact of increased volumes of antibiotics from prescriptions in humans being released in the environment [3,51]	• Gain in public and political attention for all threats related to communicable diseases, including already endemic issues such as AMR • Possible decrease in antimicrobial consumption in animals because of reduction in the size of livestock herds [52], possibly combined with difficulties in obtaining antibiotics

AMR: antimicrobial resistance; CI: confidence interval; COVID-19: coronavirus disease; HCW: healthcare worker; ICU: intensive care unit; IPC: infection prevention and control; MDRO: multidrug-resistant organism; PPE: personal protective equipment; SARS-CoV-2: severe acute respiratory syndrome coronavirus 2; WHO: World Health Organization.

## Keeping the momentum

On the occasion of European Antibiotic Awareness Day (EAAD), the European Centre for Disease Prevention and Control (ECDC) will publish its annual reports on surveillance of AMR from the European Antimicrobial Resistance Surveillance Network (EARS-Net) and on surveillance of antimicrobial consumption in humans from the European Surveillance of Antimicrobial Consumption Network (ESAC-Net). Data up to 2019 are already available from the ECDC Surveillance Atlas of Infectious Diseases [20] and the ESAC-Net database [22]. The World Health Organization (WHO) Regional Office for Europe will also publish an update of its annual report of the Central Asian and European Surveillance of Antimicrobial Resistance (CAESAR) [23], including data up to 2019. The European Medicines Agency (EMA) just published its 10th report on the European Surveillance of Veterinary Antimicrobial Consumption with data from 2018 [24]. ECDC, EMA and the European Food Safety Authority are currently working on a third Joint Interagency Antimicrobial Consumption and Resistance Analysis report, to be published in 2021, which will include a detailed One Health analysis based on data from 2016 to 2018. At the global level, the latest WHO Global Antimicrobial Resistance Surveillance System Early Implementation Report 2020 on AMR has data from 2018 [25].

Unfortunately, more recent data are not yet available from these networks to assess the impact of COVID-19 on AMR. As of December 2018, only nine EU/EEA countries used machine-to-machine links for reporting data from clinical laboratory information management systems to a national database on EU-notifiable diseases and on AMR, respectively [26]. While the collection and analysis of certain data requires time, we obviously need to implement systems for AMR surveillance in Europe that can obtain data and provide results much faster, taking as examples those that have already been implemented or are being tested in some European countries or projects [26,27].

In addition, specific studies will need to be performed to assess changes in antibiotic prescribing, IPC practices and their effect on AMR as a consequence of the COVID-19 pandemic. One important point that will require consideration is that the effects of changes in antibiotic prescribing and IPC practices on AMR is unlikely to be immediate, and that the necessary delays to observe these effects—as well as the thresholds above which changes in antibiotic prescribing and IPC result in an effect—will need to be taken into account when analysing data [28]. We should learn from the COVID-19 pandemic, with its rapid turnover and use of whole genome sequencing data, and dynamic linkage of various health databases [8,29]. Surveillance data based on clinical samples in particular, if focused only on bloodstream infections, may provide a distorted picture of the effect of COVID-19 on AMR since the effect may only manifest itself in other clinical sites or even only in the commensal flora. This means that studies based on existing surveillance data should be complemented by well-designed cohort studies with serial surveillance swabs, e.g. in ICUs and other high-risk units.

## European Antibiotic Awareness Day and World Antimicrobial Awareness Week 2020

On 18 November, we will celebrate the 13th EAAD, in partnership with the World Antimicrobial Awareness Week, from 18 to 24 November 2020. Much has happened in Europe since the first EAAD in 2008: repeated awareness campaigns took place, EU and national action plans were developed in most EU/EEA countries and regulatory actions, policy initiatives and interventions were enacted at EU and national levels. One example of EU regulatory action is provided in this issue by Opalska et al., who reviewed all EU post-authorisation procedures of harmonising product information for antibiotics from 2007 to 2020. The study found that the majority resulted in a restriction of indications for antibiotics, which could have contributed to decreasing their consumption and, ultimately, AMR [30]. The authors are planning further studies on the effect of such regulatory actions on antimicrobial consumption to inform future policies.

As suggested last year by Peñalva et al., there are signs that the many actions and initiatives implemented at EU and national levels may start to show their effects on antimicrobial consumption and AMR trends [31]. Nevertheless, much remains to be done to prevent and control AMR in Europe. The most recent data from EARS-Net confirm that AMR is still a serious challenge for the EU/EEA. In particular, the percentages of *Enterococcus faecium* from bloodstream infections that are resistant to vancomycin almost doubled between 2015 and 2019. Resistance to carbapenems—a last-line group of antibiotics—continues to be a concern, with several countries reporting carbapenem-resistance proportions above 10% in *Klebsiella pneumoniae*, and very much higher in *Pseudomonas aeruginosa* and *Acinetobacter* species bloodstream infections [20]. In this issue, a survey by Lötsch et al. provides further evidence on *A. baumannii*. Authors found that seven European countries report an endemic situation, while another nine report regional or inter-regional spread of specifically carbapenem-resistant *A. baumannii*, with national capacities for its surveillance and containment varying depending on the country [32]. For example, only 23 of the 37 participating European countries had a surveillance system for reporting carbapenem-resistant *A. baumannii*, 15 had national recommendations or guidelines for its control and only eight countries had a national plan for its containment—only one of which was one of the seven endemic countries.

The COVID-19 pandemic reminds us that compliance with IPC measures is critical to ensure the safety of hospitalised patients. Most IPC measures that are essential for controlling the spread of SARS-CoV-2 also contribute to reducing the spread of antimicrobial-resistant bacteria; these, together with antimicrobial stewardship programmes, must be maintained and strengthened. In the midst of the COVID-19 pandemic, we certainly must not give up on our efforts to prevent and control AMR and must stay united to preserve the effectiveness of antimicrobials.
